# Self-puberty staging in endocrine encounters during the COVID pandemic

**DOI:** 10.3389/fendo.2024.1487329

**Published:** 2024-10-28

**Authors:** Chineze Ebo, Jordyn McCray, Katherine Bowers, Susan R. Rose, Nana-Hawa Yayah Jones

**Affiliations:** ^1^ Divisions of Endocrinology, Cincinnati Children’s Hospital Medical Center and University of Cincinnati College of Medicine, Cincinnati, OH, United States; ^2^ Divisions of Biostatistics & Epidemiology, Cincinnati Children’s Hospital Medical Center and University of Cincinnati College of Medicine, Cincinnati, OH, United States

**Keywords:** puberty, self-staging, telemedicine, COVID, tanner stage

## Abstract

**Background:**

Evaluation of pubertal development is crucial in Endocrinology. The rise in telemedicine during COVID-19 has made conduct of physical examinations more challenging, especially for pubertal assessment. Previous studies on validity of pubertal self-staging versus clinical examination have yielded mixed results. This study aimed to determine validity and reliability of self-staging of puberty, with potential application during telemedicine visits. The present study is the first to assess pediatric self-rated pubertal staging during the pandemic.

**Methods:**

The study included patients aged 7-22 years referred to Pediatric Endocrinology for specialty care, including pubertal staging. At clinic check-in, patients received a packet with study description, an option to “opt in” or “opt out”, sex-specific self-staging instructions, and Tanner (T) stage illustrations. Males received materials for pubic hair (PH) stages T1-T5; females received materials for PH and breast (BR) stages T1-T5. Patients who opted in had 10 minutes to select the image(s) that best matched their bodies, which they sealed in an envelope. This was followed by a clinic visit, where a board-certified pediatric endocrinologist conducted a physical examination, including breast staging (females), testicular size measurement (males), and pubic hair staging (both sexes). Pubertal stage Kappa statistics with 95% CI were calculated for each body part by sex, with Kappa ≥ 0.60 indicating significant agreement between self-assessment and physician assessment (0.40-0.60 moderate; 0.20-0.40 fair).

**Results:**

Of 516 distributed packets, 243 self-assessments (125 females) were returned, with 81% (94 females/102 males) being complete (including pediatric endocrinologist staging). Mean age of participants was 12.8 years. Mean BMI was 22.2 kg/m² (males) and 23.7 kg/m² (females). Hypothyroidism was the most common endocrine diagnosis. For females, kappa was highest for BR and PH in T1 (BR 0.65, PH 0.57) and T5 (BR 0.57, PH 0.65). For males, kappa was highest in T1 (0.73) and T2 (0.58). Grouping Tanner stages into prepuberty (T1), early to mid-puberty (T2-T3), and late puberty (T4-T5) showed greater agreement.

**Conclusion:**

Patients can reliably distinguish between “puberty” and “no puberty” using self-staging, though differentiating between later pubertal stages is more challenging. These findings help define the utility and limitations of self-staging during telemedicine visits.

## Introduction

Puberty represents a critical period of development characterized by important hormonal, physical, and cognitive changes, with direct influences on overall body physiology and growth. This period significantly impacts overall Pediatric Endocrine care, as the increase of sex steroids (estrogen or testosterone) drives the development of secondary sexual characteristics and influences metabolic and hormonal parameters (1, 2). Moreover, these hormonal shifts extend beyond sexual maturation, affecting multiple systems throughout the body. From metabolism and growth to thyroid regulation, respiratory, and neurological functions, the broad impact of puberty underscores its importance in shaping overall health during this critical developmental stage (3–7) Thus, accurate pubertal staging and monitoring of puberty are essential for early detection of abnormalities and for supporting healthy development in children and adolescents.

Tanner staging is a physical examination method used to assess pubertal development in children, adolescents, and adults, as initially described by Marshall and Tanner (8). This method categorizes the puberty of individuals into stages based on the development of secondary sexual characteristics such as breast development, pubic hair growth, and genital development. The gold standard practice of Tanner staging involves documentation by a trained clinician (9). Classically, Tanner Stages range from Stage 1 (prepubertal) to Stage 5 (adult development), providing an important framework for diagnosing disorders of puberty, including delayed or precocious puberty. Failure to diagnose delayed puberty can lead to reduced adult height, low bone density with a higher risk of osteoporosis, and increased susceptibility to cardiovascular and metabolic disorders later in life (10). Conversely, undiagnosed precocious puberty can lead to short stature, reproductive issues, increased cardiometabolic risk, and psychological challenges associated with early sexual development (11).

In 2020, the global coronavirus disease-19 pandemic, also known as COVID-19, changed the standards of medical practice. While quarantine measures helped reduce transmission of the disease, they also posed significant challenges to physicians’ ability to deliver adequate, timely, and effective health care. To address these challenges, telemedicine gained popularity, offering patients healthcare access while minimizing exposure for vulnerable populations (12) The COVID-19 pandemic served as an opportunity to adopt teleconsultation in response to the urgent need for continuity of care and has indefinitely led to reimagining its use in the management of both acute and chronic diseases (13, 14). In pediatric endocrinology, physical examination of the genitourinary system is crucial; however, telemedicine is an inappropriate platform for this part of the physical examination and does not allow for accurate assessment of common endocrine evaluations, such as pubertal staging.

The rise of telemedicine has made it imperative to find a way to conduct clinical assessments of puberty, as neglecting them could result in harm. This raises an important question: how accurate and reliable are patient self-assessments of puberty in this digital age? Previous research has documented the ability of children and adolescents to self-assess their pubertal development using various tools. These tools have included questionnaires with only written descriptions, those supplemented by drawings (with or without additional descriptions), and those that incorporate real-life photography (with or without written descriptions) (15–19). A number of studies have explored the accuracy of self-staging puberty compared to clinical examinations, noting that results can vary based on the patient’s age, self-assessment skills, race, and cultural background (20, 21). Overall, the literature presents inconclusive views on the validity of these self-assessments.

There are clear challenges with using telemedicine for physical evaluations, particularly in pediatric endocrinology where monitoring growth and physical development is important. The aim of this study was to determine validity and reliability of patients’ self-staging pubertal assessments for potential use at home during telemedicine visits. To our knowledge, this study is the first to evaluate self-rated pubertal staging by children and adolescents during the COVID-19 pandemic.

## Materials and methods

This was a cross-sectional study conducted over 13 months (October 2021-November 2022) at Cincinnati Children’s Hospital Medical Center (CCHMC), involving pediatric patients ages 7-22 years who were referred to our tertiary pediatric endocrinology center The study received approval from the Institutional Review Board, and verbal parental consent along with participant assent was obtained. The inclusion criteria focused on those patients for whom pubertal assessment was deemed necessary by their Pediatric Endocrinologist. Exclusion criteria included a known diagnosis of cognitive developmental delay or gender dysphoria.

Upon clinic check-in, eligible patients and/or their guardians received a study packet from an Endocrine nurse containing several key documents: a detailed explanation of the study’s procedures, objectives, and rationale; a section allowing patients to voluntarily “opt in” or “opt out” of the study; and written, sex-specific instructions on performing self-pubertal staging. These instructions were accompanied by gender-appropriate illustration sheets depicting the Tanner stages (T1-T5) for both pubic hair (PH) and, for girls, breast development (BR) (17) [**Supplementary Materials 1–4**]. Of note, male participants were not asked to self-stage testicular size. Those who opted to participate marked “opt in” on the informational sheet and informed the nurse, who then escorted them to the clinic room with their packet.

Patients completed the self-assessment in the absence of the healthcare provider. They had up to 10 minutes to review the illustrations and self-assess their pubertal stage by selecting the Tanner Stage illustration that they felt most closely resembled their current stage of pubertal maturation. Patients then marked their selections on the provided papers, which were then sealed in an envelope with the patient’s medical record number (MRN) for later correlation with clinician results. The sealed envelope was handed to the nurse, who placed it in a secure location for delivery to the study team.

Following the self-assessment phase, participants underwent a standard clinic visit, with a complete physical examination by a board-certified pediatric endocrinologist including breast staging for girls, and pubic hair assessment for both sexes. The physical examination took place on the same day as the clinic visit.

For data retrieval, the patient’s clinic visit, including the physical examination by the endocrinologist, was accessed from the electronic medical record system using the patient MRN on the envelope. Study data were collected and managed using REDCap electronic data capture tools hosted at CCHMC (22, 23). The recorded data included date of clinic appointment, date of birth, calculated age, sex, race or ethnicity, weight (in kg) with percentile and z-score for age, height (in cm) with percentile and z-score for age, body mass index, any comorbidities or relevant past medical history, medications, self-assessed breast score (for females), self-assessed pubic hair score (for both females and males), physician-assessed Tanner stage for breast development (for females), and physician-assessed Tanner stage for pubic hair (for both females and males).

All demographic information and any patient identifiers were kept in a secure location and only accessible to those involved with the study. After all data were entered in REDCap, the envelope was discarded. To ensure privacy and confidentiality, patients were identified only by numbers in REDCap.

Statistical analyses were conducted using SAS version 9.4 (SAS Institute Inc., Cary, NC). Prior study results were used to estimate the required sample size for significance (17). We would require at least 80 participants of each sex to achieve significance, preferably with 16 participants within each Tanner Stage.

Baseline characteristics of the study population were described using means and standard deviations for continuous variables, and percentages for categorical variables. These descriptive statistics were reported for the overall population as well as separately for each sex.

Agreement between self-staging and endocrinologist staging was assessed using the weighted kappa statistic, with 95% confidence intervals (CI) calculated for each sex and body site and stratified by Tanner Stage. Additionally, a pubertal stage kappa statistic was computed to evaluate agreement between self-staging and endocrinologist staging for breast and pubic hair development, with separate 95% CIs for each sex. Kappa values were interpreted as follows: ≥0.6 indicated substantial agreement, 0.4-0.6 indicated moderate agreement, and 0.2-0.4 indicated fair agreement (24). Additionally, we conducted a separate analysis using a simplified grouping of pubertal stages, categorizing them as pre-puberty (Tanner Stage 1), early to mid-puberty (Tanner Stages 2 and 3), and late puberty (Tanner Stages 4 and 5). This approach aimed to assess whether the accuracy of self-staging could be enhanced by utilizing broader pubertal stage categories.

## Results

### Participant demographics and clinical characteristics

From the 516 packets distributed, 243 self-assessments were received, representing a response rate of 47.1%. Of them, 196 (80.6%) were deemed complete, inclusive of both self-reported pubertal assessments and endocrinologist Tanner staging, with gender representation remaining relatively balanced (48.0% females) (**Table 1**). The mean (± SD) age of participants at the time of clinical examination was 12.5 ± 2.8 years, age 11.8 ± 3.3 years for females and 13.1 ± 2.1 years for males (**Table 2**). Notably, analysis of body mass index (BMI) revealed discernible gender differences, with males displaying a mean (± SD) BMI of 21.9 ± 6.4 kg/m^2, while females exhibited a slightly higher mean BMI of 22.7 ± 7.4 kg/m^2. Of all respondents who had a complete endocrinologist staging, 46.4% had high BMI (greater than or equal to the 85th percentile), with 45.1% (n=46) of males and 47.9% (n=45) of females falling into this category.

**Table 1:** Self-Assessments of breast and pubic hair development (tanner stages 1–5) versus endocrinologist tanner staging.

Endocrinologist Tanner Staging	Self-Assessment N (%)					Total *N (%)*
	1	2	3	4	5	
Breast stage						
1	11 (11.7)	6 (6.4)	0 (0)	0 (0)	0 (0)	17 (18.1)
2	0 (0)	11 (11.7)	1 (1.1)	2 (2.1)	0 (0)	14 (14.9)
3	3 (3.2)	12 (12.8)	14 (14.9)	2 (2.1)	0 (0)	31 (32.9)
4	0 (0)	1 (1.1)	3 (3.2)	3 (3.2)	1 (1.1)	8 (8.5)
5	0 (0)	0 (0)	2 (2.1)	10 (10.6)	12 (12.8)	24 (25.5)
Total	14 (14.9)	30 (31.9)	20 (21.3)	17 (18.1)	13 (13.8)	94 (100)
Females						
Pubic hair stage						
1	11 (11.7)	5 (5.3)	2 (2.1)	1 (1.1)	0 (0)	19 (20.2)
2	4 (4.3)	11 (11.7)	4 (4.3)	0 (0)	0 (0)	19 (20.2)
3	0 (0)	7 (7.5)	6 (6.4)	7 (7.5)	0 (0)	20 (21.3)
	0 (0)	1 (1.1)	2 (2.1)	5 (5.3)	1 (1.1)	9 (9.6)
5	0 (0)	1 (1.1)	0 (0)	10 (10.6)	16 (17.0)	27 (28.7)
Total	15 (16.0)	25 (26.6)	14 (14.9)	23 (24.5)	17 (18.1)	94 (100)
Males						
Pubic hair stage	1	2	3	4	5	
1	27 (26.4)	11 (10.9)	0 (0)	0 (0)	0 (0)	38 (37.2)
2	0 (0)	17 (16.8)	2 (2.0)	1 (1.0)	0 (0)	20 (19.8)
3	0 (0)	2 (2.0)	8 (7.9)	3 (3.0)	4 (4.0)	17 (16.8)
4	0 (0)	0 (0)	1 (1.0)	10 (9.9)	4 (4.0)	15 (14.9)
5	0 (0)	0 (0)	0 (0)	5 (5.0)	7 (6.9)	12 (11.9)
Total	27 (26.4)	30 (29.7)	11 (10.9)	19 (18.8)	15 (14.9)	102 (100)

**Table 2:** “Opt in” study population characteristics.

	Males (n=102)	Females (n=94)
	Mean ± Standard Deviation	
Age, years	13.1 ± 2.1	11.8 ± 3.3
Height, cm	151.1 ± 14.9	146.6 ± 14.5
Weight, kg	51.6 ± 22.1	50.9 ± 24.4
BMI, kg/m2	21.9 ± 6.4	22.7 ± 7.4
BMI Percentile	60.1 ± 37.1	70.0 ± 30.7
BMI >85th percentile (%)	45.1%	47.9%
Most Prevalent Primary Endocrine Diagnoses Among All Sexes (%)*		
Thyroid Disorders	34.3	
Growth Disorders	31.6	
Pubertal Disorders	28.3	
Obesity-related Disorders	16.9	

*Many patients had more than 1 diagnosis.

The majority of participants in this study were White (79%), followed by Black or African American (14%) and Asian (4%); the remaining 3% were Hispanic, Native Hawaiian or Other Pacific Islander, American Indian or Alaska Native, or identified as Other. Participants were being seen in the endocrine clinic for their primary endocrine diagnoses (many had more than one endocrine diagnosis). Thyroid disorders (34.3%) and growth disorders (31.6%) were the most prevalent diagnosis, followed by pubertal disorders (28.3%) and obesity-related conditions (16.9%). Other less common diagnoses included adrenal disorders, bone and calcium disorders, hypopituitarism and others. Notably, some participants also had additional co-morbidities, including mood disorders (16.3%), neurologic disorders (15.9%), and ADHD/attention disorders (13.4%). The top three medications prescribed were for thyroid disorders (29.5%), mood/behavior/development disorders (20.2%), and growth disorders (11.8%).

### Agreement in pubertal staging between physician and patient

**Figure 1:**
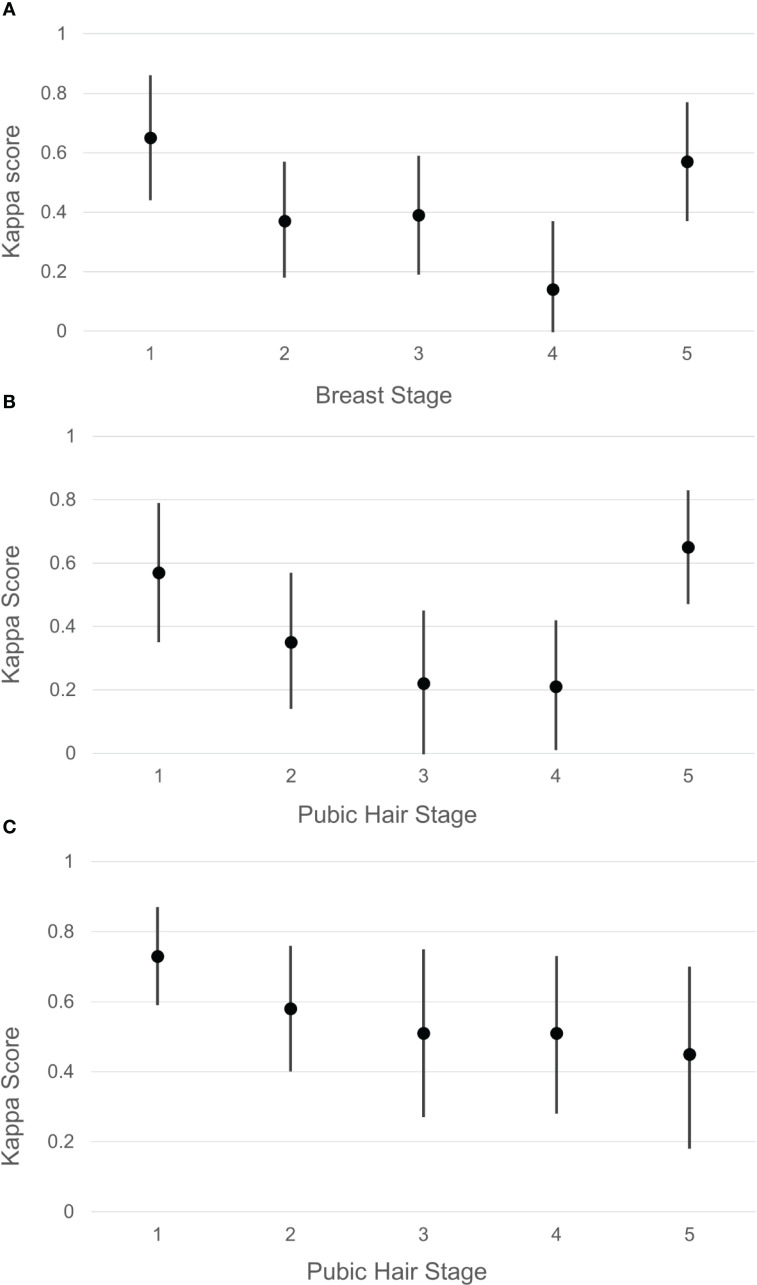
Agreement (represented by Kappa score and 95% confidence interval) between pubertal self-staging and pediatric endocrinologist assessment by Tanner stage for patients seen in endocrinology clinic. **(A)** Female breast stage. **(B)** Female pubic hair. **(C)** Male pubic hair.

The distribution of agreement between endocrinologists’ assessment and self-assessment are shown in **Table 1**. Among females, the highest level of agreement with endocrinologists was observed at Tanner stage 1 (T1) for breast development (kappa = 0.65, 95% CI 0.44-0.86) (**Figure 1A**) and pubic hair development (kappa = 0.57, 95% CI 0.35-0.79) (**Figure 1B**), as well as at Tanner stage 5 (T5) for both breast (kappa = 0.57, 95% CI 0.37-0.77) and pubic hair (kappa = 0.65, 95% CI 0.47-0.83). Conversely, the lowest level of agreement with endocrinologists was observed at Tanner stage 2 (T2) for breast (kappa = 0.37, 95% CI 0.18-0.57) and Tanner stage 4 (T4) for breast (kappa = 0.14, 95% CI –0.09-0.37). Interestingly, higher BMI percentile (>85th percentile) was associated with higher kappa scores for breast (kappa = 0.74, 95% CI 0.63-0.86) and pubic hair (kappa = 0.71, 95% CI 0.60-0.84) compared to lower BMI percentile (< 85th percentile) for breast (kappa = 0.52, 95% CI 0.37-0.66) and pubic hair (kappa = 0.58, 95% CI 0.44-0.72).

Among males, the highest level of agreement with endocrinologists was observed at Tanner stage 1 (kappa = 0.73, 95% CI 0.59-0.87) (**Figure 1C**) and Tanner stage 2 (kappa = 0.58, 95% CI 0.59-0.87) for pubic hair. Conversely, the lowest level of agreement was observed at Tanner stage 5 pubic hair (kappa = 0.45, 95% CI 0.18-0.70). The same kappa agreement was observed at both Tanner 3 and 4 pubic hair (kappa = 0.51). In contrast to the pattern observed in females, higher BMI percentile was associated with lower kappa scores (kappa = 0.69, 95% CI 0.56-0.80) for pubic hair compared to lower BMI percentile (kappa = 0.82, 95% CI 0.73-0.91).

**Figure 2:**
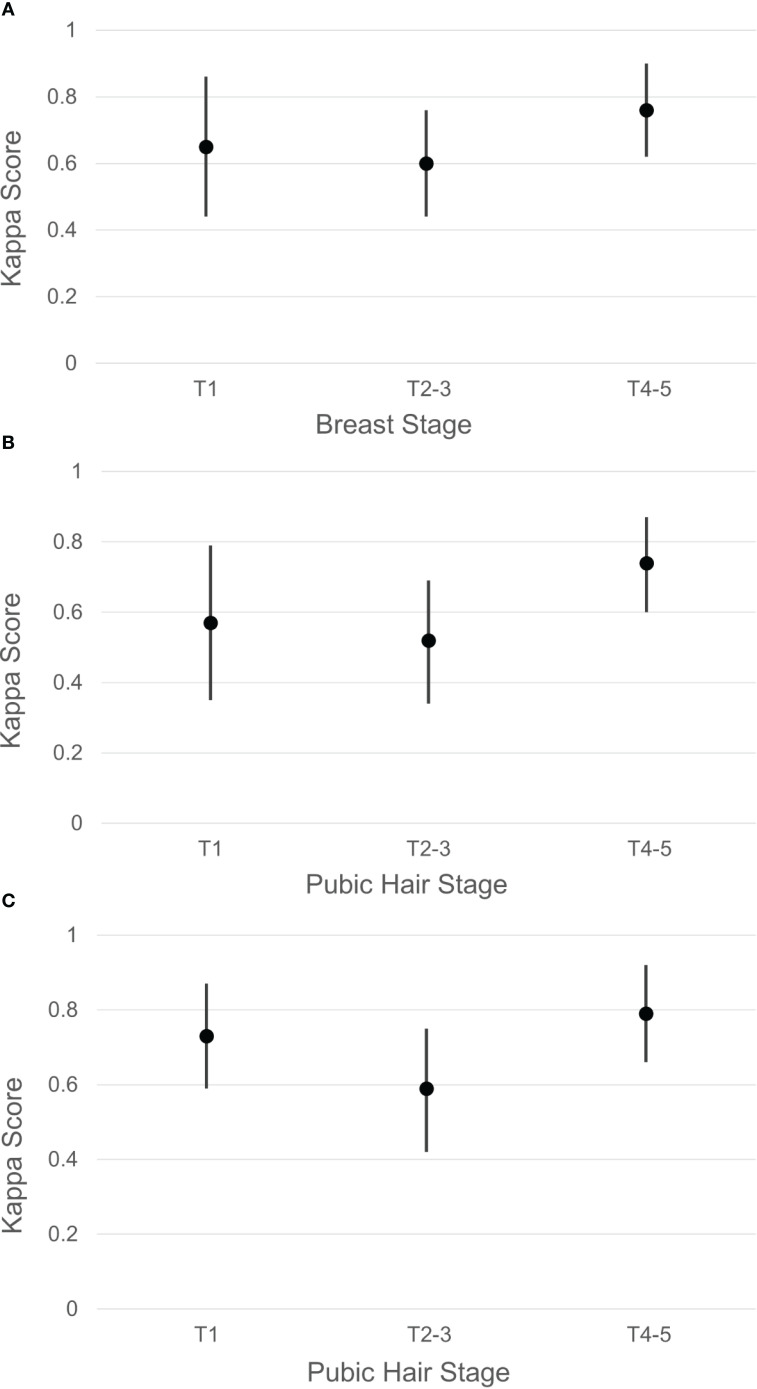
Agreement (represented by Kappa score and 95% confidence interval) between pubertal self-staging and pediatric endocrinologist assessment by grouped Tanner stages (pre-puberty T1, early to mid-puberty T2 to T3, or late puberty T4 to T5) for patients seen in endocrinology clinic. **(A)** Female breast stage. **(B)** Female pubic hair. **(C)** Male pubic hair.

When examining agreement of physician Tanner Staging and self-staging of puberty categorized as pre-puberty (T1), early to mid-puberty (T2-T3), and late puberty (T4-T5), we observed a notable increase in agreement levels. Specifically, during pre-puberty (T1), breast development in females exhibited a kappa of 0.65 (**Figure 2A**), while pubic hair development showed a kappa of 0.57 for females (**Figure 2B**) and 0.73 for males (**Figure 2C**). In early-mid puberty (T2-T3), the kappa for breast was 0.60, with pubic hair at 0.52 for females and 0.59 for males. In late puberty (T4-T5), agreement for breast was higher (kappa 0.76), with kappa for pubic hair reaching 0.74 for females and 0.79 for males.

## Discussion

This study compared self-staging of puberty by children and adolescent patients with assessments made by a pediatric endocrinologist, and evaluated accuracy of self-assessments in pubertal development. The primary goal was to determine the validity and reliability of self-staging of puberty for potential use during telemedicine visits.

Our study found that among females, the highest levels of agreement with endocrinologists were observed at the initial (Tanner stage 1) and final (Tanner stage 5) stages of breast and pubic hair development. This indicates that at the very beginning and end of puberty, females are more capable of accurately self-assessing their development. In contrast, the lowest agreement was noted during Tanner stages 2 and 4 for breast development, suggesting increased difficulty in self-assessment during the intermediate stages of puberty. For males, the highest agreement with endocrinologists was seen at Tanner stages 1 and 2 for pubic hair development, similar to females, indicating that self-assessment appears to be most reliable at the onset of pubertal maturation. Conversely, for males the lowest agreement was observed at Tanner stage 5, with intermediate stages (Tanner 3 and 4) showing moderate agreement levels.

When examining agreement levels by broader pubertal categories, we found an overall improvement in agreement when grouping Tanner stages into pre-puberty (T1), early to mid-puberty (T2-T3), and late puberty (T4-T5). Specifically, pre-puberty stages showed improved agreement for both breast and pubic hair development across genders. Agreement levels remained moderate during early to mid-puberty, and significantly increased during late puberty. These findings indicate that a broader categorization may simplify the self-assessment process and improve its accuracy. Additionally, the data suggest a consistent pattern of increasing agreement during the later stages of puberty, highlighting a trend towards greater concordance as pubertal maturation progresses.

Interestingly, the current study found that a higher BMI percentile was associated with higher agreement scores for both breast and pubic hair development among females. This was surprising to us, as prior research suggested that elevated BMI and higher levels of body fat can hinder the ability to differentiate between lipomastia and true breast tissue, which may result in an exaggerated assessment of breast development (25). This is also in contrast with previous literature that found that BMI did not appear to significantly influence self-assessment (16). Notably, the earlier study featured a higher proportion of female participants with lower weights and BMIs, whereas nearly 50% of the females in our study had BMIs above the 85th percentile. This difference in BMI distribution may explain the discrepancy between our findings and those of the earlier research. Nonetheless, it appears that accuracy was not confounded by being overweight, indicating that self-assessment reliability can still be achieved across different BMI percentiles. In contrast, males with higher BMI percentiles exhibited lower agreement scores for pubic hair development. We did not assess gonadarche in males and thus we do not know if self-assessment of gonadarche is confounded by being overweight in male patients. Further research is justified.

There are some limitations affecting this study. Not all individuals who self-evaluated their Tanner Stage received a “gold standard” physical assessment by a pediatric endocrinologist. Specifically, for 47 patients, no Tanner stage examination was documented in the electronic medical record. It remains unclear whether the exam was not conducted, not documented, or declined by the patient. Additionally, the endocrinologist may have determined that the pubertal exam was not clinically relevant for that specific visit. As a result, we may have missed possible instances of concordance or discordance between self-assessments and expert appraisals. There was also an unequal distribution and representation of Tanner stages among participants, which may have impacted the generalizability of our findings. Finally, male participants were not asked to self-assess testicular size, which is an integral component of accurately evaluating pubertal status. While we considered including testicular measurements, we determined that it could impose additional burden on both participants and the study process. Collecting such data might have introduced complexity and extended the duration of examinations, potentially affecting participant compliance and the overall feasibility of the study. Therefore, we made the decision to focus on other markers of pubertal maturation that could be more readily and consistently measured across participants using visual, not tactile, cues. However, this omission may have resulted in incomplete or inaccurate assessments of male pubertal development.

Despite these limitations, our study has several strengths. A key strength was that the gold standard physical examination was conducted by a pediatric endocrinologist who was blinded to the patient’s self-assessment. Both the physician examination and the patient’s self-staging were performed on the same day, ensuring consistency. Additionally, a large cohort of patients submitted self-assessments, providing robust data. Lastly, the study population was diverse, representing a range of racial backgrounds, primary endocrine diagnoses, and anthropometric measurements.

## Conclusion

We compared self-staging of puberty by children and adolescent patients with the Tanner stage physical examination made by a pediatric endocrinologist, evaluating the accuracy of self-assessments in pubertal maturation. The findings indicate that, in general, children and adolescents can accurately distinguish between “puberty” and “no puberty” using self-staging, although differentiating between individual pubertal stages is less reliable. A greater level of agreement was observed when female and male Tanner stages were grouped into pre-puberty, early to mid-puberty, and late puberty categories. Thus, pubertal self-staging can potentially serve as a valuable and efficient clinical tool, offering a viable alternative to in-person physical examinations, particularly when patients are unable to attend clinic visits. Consequently, this study contributes to understanding the utility and limitations of self-staging during telemedicine visits, highlighting its potential role in clinical practice particularly as telehealth continues to be an important part of healthcare post-pandemic (26).

## Data Availability

The original contributions presented in the study are included in the article/**Supplementary Material**. Further inquiries can be directed to the corresponding author.
