# Supplementary material for: Self-puberty staging in endocrine encounters during the COVID pandemic
Source: Front Endocrinol (Lausanne). 2024 Oct 28;15:1487329. doi: 10.3389/fendo.2024.1487329 (PMC11550967; doi:10.3389/fendo.2024.1487329)
Supplement: Supplementary file 1 [file DataSheet1.pdf]

Informational sheet about Endocrine Study: Self Pubertal Staging - Validity and Reliability in Endocrine Telemedicine Encounters

Description:

You are invited to participate in a research study today during your clinic visit. Dr. Nana Jones, Dr. Chineze Ebo, and Dr. Susan Rose are conducting this study to figure out whether children can accurately check their own puberty stage by looking at his/her/their height, weight, and puberty. It is important to know your child's stage of puberty because this can affect growth and development, which is very important in Endocrine care. Children go through puberty at different times and at different rates. There are no right or wrong answers. Every individual child is different. The duration of this study will be 12 months. It will take approximately 5-10 minutes for your participation today.

As part of this study, we will ask you (or your child) to stage your own puberty during your visit today. We will provide papers (one for girls and one for boys) that have drawings and descriptions on them. As you (or your child) are changing your clothes, take 5 minutes to look carefully at your body. Then look at the drawings with written descriptions that are on the pages.

Find the drawing and description that is most like your body today. Place a check mark in the box next to that drawing and description.

Participation in this study is completely voluntary - You can say yes or no without any consequence. You can choose not to participate in the study. Please let your nurse know if you/your child will accept or decline participation in this study. If you choose to participate in this study, the nurse will give you instructions on how to check your puberty stage, along with an envelope to put your answer in.

When the doctor comes in, they will still check your puberty stage during the visit as they normally would, and they will put their assessment in the chart.

Risks and Benefits:

There are minimal risks associated with this study, as you (or your child) will be examined by an Endocrine doctor today regardless of you doing this study. Your decision to participate or not participate will not affect your clinic visit, treatment, or patient care by any means. Potential benefits are that children and parents can gain better understanding of what staging puberty means. Another benefit is that the information collected in the study will provide helpful information that can help patient care in the future.

Participants' Rights:

If you have read this form and have decided to be in this study, please understand your **being in this study is voluntary** and you have the right to stop being in the study at any time without any bad effects. The results of this research study may be presented at scientific meetings or published in scientific journals. Your individual privacy will be maintained in all published and written data resulting from the study. The information collected from this study may be used for future research studies or distributed to another investigator for future studies without additional informed consent being obtained.

Contact:

Questions about this study: You can contact Chineze Ebo, MD at chineze.ebo@cchmc.org for answers to pertinent questions about the research and research participants' rights.

If you agree to participate in this research, please place a check mark in the box next to "OPT IN" below. If you do not want to participate in this research, please place a check mark in the box next to "OPT OUT" below.

OPT IN ☐ **OPT OUT** ☐
