# Supplementary material for: Self-puberty staging in endocrine encounters during the COVID pandemic
Source: Front Endocrinol (Lausanne). 2024 Oct 28;15:1487329. doi: 10.3389/fendo.2024.1487329 (PMC11550967; doi:10.3389/fendo.2024.1487329)
Supplement: Supplementary file 2 [file DataSheet2.pdf]

<u>Pubertal Staging Instruction Sheet</u>

<u>Girls</u>: Do this for the page that shows breast growth AND ALSO for the page that shows pubic hair growth. The first page shows breast growth as girls go through puberty. The second page shows pubic hair growth as girls go through puberty. The earliest stage is at the top, and the adult stage is at the bottom. As you (or your child) are changing your clothes, take 5 minutes (can be longer if needed) to look carefully at your body. Then look at the drawings with written descriptions that are on the pages. Find the drawing and description that is most like your body today. Place a check mark in the box next to that drawing and description. When you are finished, place all pages inside the envelope and seal it. You should then get dressed again. Give the envelope back to the nurse or medical assistant who is working with you today.

<u>Boys</u>: Do this for the page that shows pubic hair growth as boys go through puberty. The earliest stage is at the top, and the adult stage is at the bottom. As you (or your child) are changing your clothes, take 5 minutes (can be longer if needed) to look carefully at your body. Then look at the drawings with written descriptions that are on the pages. Find the drawing and description that is most like your body today. Place a check mark in the box next to that drawing and description. When you are finished, place all pages inside the envelope and seal it. You should then get dressed again. Give the envelope back to the nurse or doctor who is working with you today

Thank you again for your important work in our research.
