# Supplementary material for: Self-puberty staging in endocrine encounters during the COVID pandemic
Source: Front Endocrinol (Lausanne). 2024 Oct 28;15:1487329. doi: 10.3389/fendo.2024.1487329 (PMC11550967; doi:10.3389/fendo.2024.1487329)
Supplement: Supplementary file 3 [file DataSheet3.pdf]

Breast Development

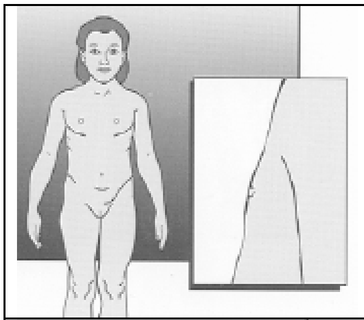	No change in size of the breast.	This looks most like my body today. ☐ BR1
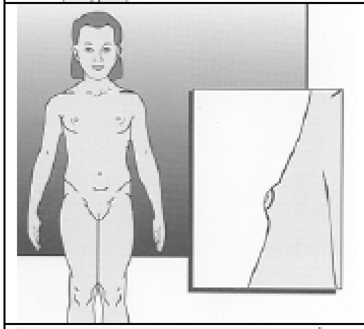	A small mound is formed; nipple size is larger.	This looks most like my body today. ☐ BR2
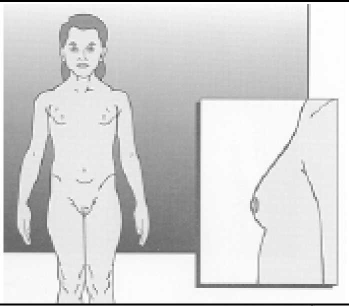	Breast and nipple are larger.	This looks most like my body today. ☐ BR3
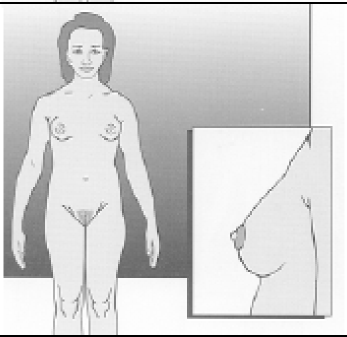	Nipple and brown area are raised above the rest of the breast.	This looks most like my body today. ☐ BR4
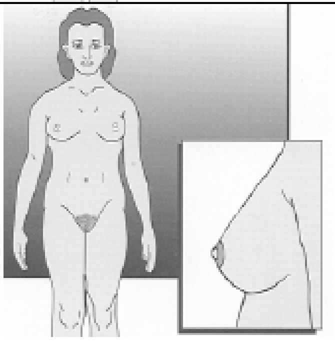	Breast looks like that of an adult; brown area is flattened to match the breast; overall increase in size of breast.	This looks most like my body today. ☐ BR5

Pubic Hair Growth

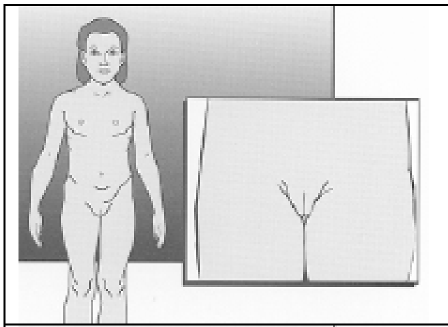	No pubic hair.	This looks most like my body today. ☐ PH1
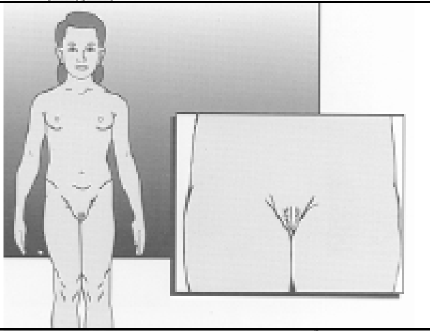	Small amount of hair (sparse, straight), light in color, mostly along the inner folds.	This looks most like my body today. ☐ PH2
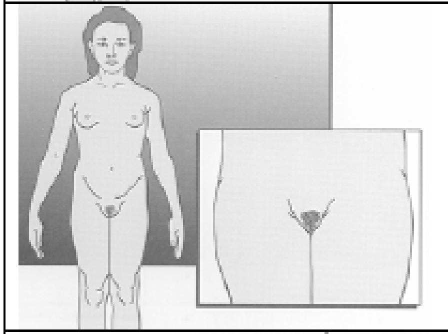	Hair is coarser, darker, and more curled, and spreads over the middle but can see skin through the sets of hairs.	This looks most like my body today. ☐ PH3
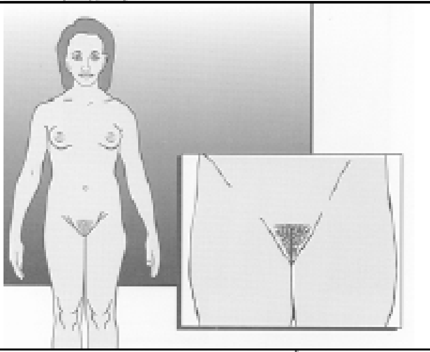	Hair looks like that of an adult but has not spread to the thighs.	This looks most like my body today. ☐ PH4
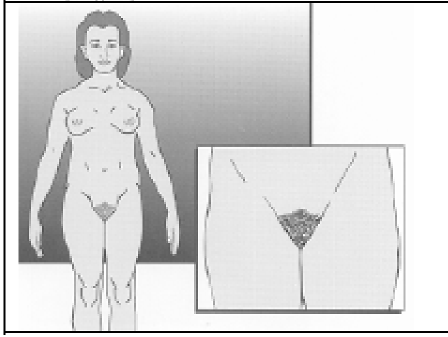	Hair looks like that of an adult and spreads to the thighs. Hair is in the shape of broad based triangle.	This looks most like my body today. ☐ PH5
