# Supplementary material for: Self-puberty staging in endocrine encounters during the COVID pandemic
Source: Front Endocrinol (Lausanne). 2024 Oct 28;15:1487329. doi: 10.3389/fendo.2024.1487329 (PMC11550967; doi:10.3389/fendo.2024.1487329)
Supplement: Supplementary file 4 [file DataSheet4.pdf]

Pubic Hair Growth

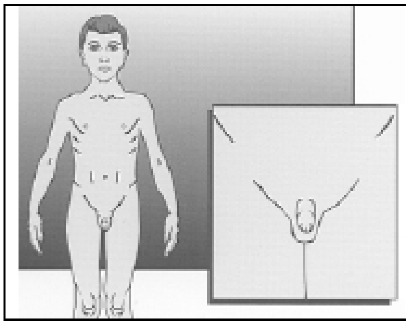	No pubic hair.	This looks most like my body today. ☐ PH1
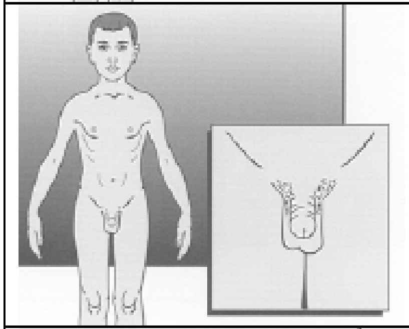	Small amount of hair (sparse, straight) mostly along the side of the penis.	This looks most like my body today. ☐ PH2
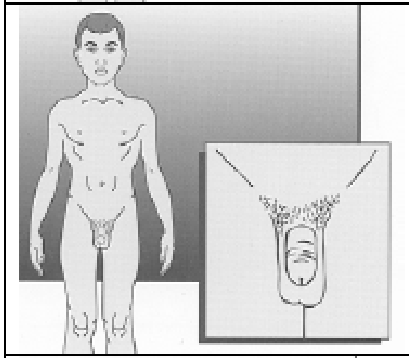	Hair is coarser, darker, and more curled, and spreads above the penis.	This looks most like my body today. ☐ PH3
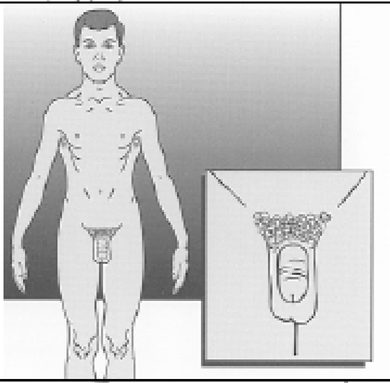	Hair looks like that of an adult, but has not spread to the thighs.	This looks most like my body today. ☐ PH4
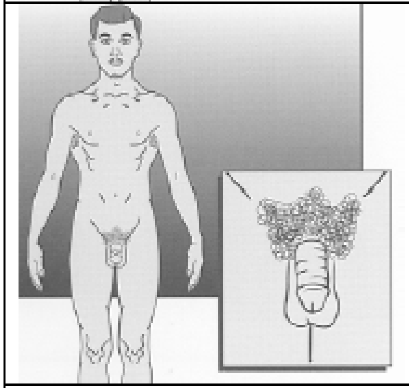	Hair looks like that of an adult and spreads to the thighs.	This looks most like my body today. ☐ PH5
